# Recovery of silver residues from dental amalgam

**DOI:** 10.1590/S1678-77572010000200005

**Published:** 2010

**Authors:** Heloísa Aparecida Barbosa da Silva PEREIRA, Flávia Godoy IANO, Thelma Lopes da SILVA, Rodrigo Cardoso de OLIVEIRA, Manoel Lima de MENEZES, Marília Afonso Rabelo BUZALAF

**Affiliations:** 1 Graduate student, Department of Biological Sciences, Bauru School Of Dentistry, University of São Paulo, Bauru, SP, Brazil.; 2 Pharm, Department of Biological Sciences, Bauru School of Dentistry, University of São Paulo, Bauru, SP, Brazil.; 3 BSc, MSc, Department of Biological Sciences, Bauru School of Dentistry, University of São Paulo, Bauru, SP, Brazil.; 4 DDS, MSc, PhD, Associate Professor, Department of Biological Sciences, Bauru School of Dentistry, University of São Paulo, Bauru, SP, Brazil.; 5 Chem, MSc, PhD, Associate Professor, Department of Chemistry, School of Sciences, State University of São Paulo, Bauru, SP, Brazil.; 6 DDS, MSc, PhD, Full Professor, Department of Biological Sciences, Bauru School of Dentistry, University of São Paulo, Bauru, SP, Brazil.

**Keywords:** Dental amalgam, Silver, Solid wastes, Environment

## Abstract

Dental amalgam residues are probably the most important chemical residues generated from clinical dental practice because of the presence of heavy metals among its constituents, mainly mercury and silver.

**Objective:**

The purpose of this study was to develop an alternative method for the recovery of silver residues from dental amalgam.

**Material and Methods:**

The residue generated after vacuum distillation of dental amalgam for the separation of mercury was initially diluted with 32.5% HNO_3,_ followed by precipitation with 20% NaCl. Sequentially, under constant heating and agitation with NaOH and sucrose, the sample was reduced to metallic silver. However, the processing time was too long, which turned this procedure not viable. In another sequence of experiments, the dilution was accomplished with concentrated HNO_3_ at 90ºC, followed by precipitation with 20% NaCl. After washing, the pellet was diluted with concentrated NH_4_OH, water and more NaCl in order to facilitate the reaction with the reducer.

**Results:**

Ascorbic acid was efficiently used as reducer, allowing a fast reduction, thus making the procedure viable.

**Conclusion:**

The proposed methodology is of easy application and does not require sophisticated equipment or expensive reagents.

## INTRODUCTION

The unbridled development of the population and industry has led to an increase in the generation of residues^2^. The natural environment is not able to support the increasing exposure to chemical products caused by this development^6^. The acknowledgement that human intervention has been contributing to the deterioration of the natural environment has led several countries to search for alternatives for its restructuring^7^.

Regarding the residues generated in dental practice, the most concerning ones are those deriving from dental amalgam^8,9,14,16^ because this metal alloy has, among its constituents, mercury, silver, tin and copper^1,12^. The use of amalgam separators has been recommended to physically remove dental amalgam from waste water in dental clinics thus reducing the mercury emissions^9,18^.

The most abundant metal in dental amalgam is mercury^1^, and its recovery from dental amalgam has been done using vacuum distillation^10,11,13^. After recovery of mercury, however, other hazardous metals are still left^1,5^, among which, silver is the most abundant^1^. This metal is very dangerous both to aquatic and terrestrial organisms^4,6,15,21^. In humans, silver is metabolized and deposited in subcutaneous fat, and its excessive ingestion generates argyria, a cosmetic disorder^3^. In addition, silver is commonly used in the industry and its recovery could allow some financial return^3,8^.

Despite some studies have described processes to recover mercury from dental amalgam^10,13^, only one study focused on the recovery of silver using sucrose as a reducing agent^11^. In the present study, the purpose was to develop an alternative method for the recovery of silver residues from dental amalgam.

## MATERIAL AND METHODS

Dental amalgam residues were received from the dental clinics of Bauru Dental School and Hospital for Rehabilitation of Craniofacial Anomalies (HRAC), University of São Paulo, Bauru, SP, Brazil. The amalgam was initially processed by vacuum distillation for mercury removal^10,13^, generating an amount of 5,516.93 g.

### Process of silver recovery using sucrose as a reducing agent

**Figure 1:**
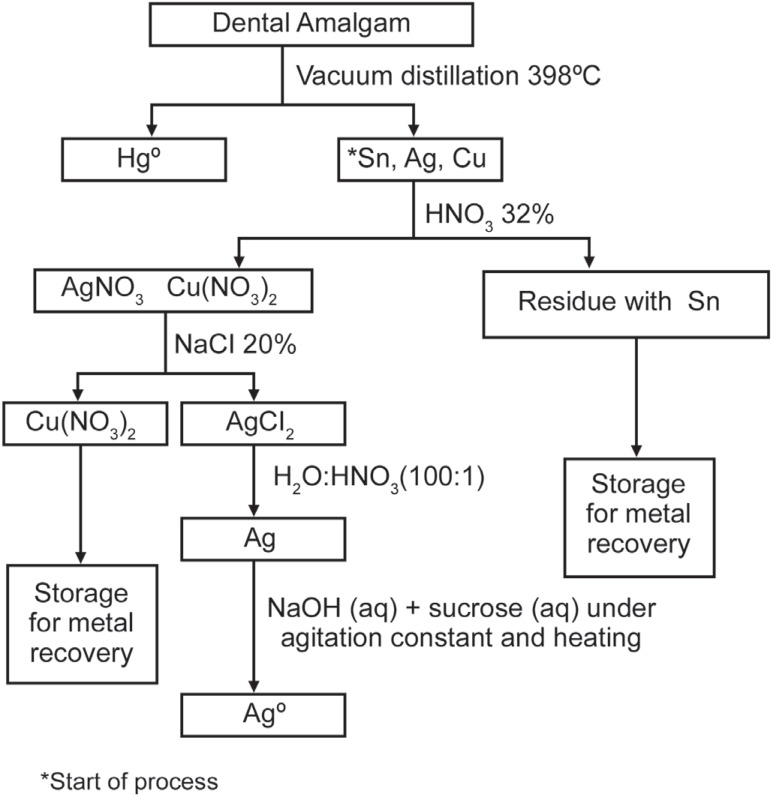
Flow chart of silver recovery using sucrose

This process was adapted from the methodology proposed by Lee and Fung^11^ (1980). The amount of samples (residues of dental amalgam after recovering mercury) used ranged between 25 and 150 g. For residue dilution, 32.5% nitric acid was used for the formation of AgNO_3_ (aq) (Table 1). The solution containing AgNO_3_ was collected in another vial, where 20% NaCl was added to promote the precipitation of silver as AgCl_2_ (solid). This pellet was washed with nitric acid and deionized water (1:100 v/v) until the supernatant became colorless. In sequence, a solution of sucrose (concentrations ranging between 2.5 and 20%) and NaOH (concentrations ranging between 10 and 40%) was added to the pellet (Table 1) and, under agitation and heating, the AgCl_2_ was reduced to Ag^0^ (Figure 1).

**Table 1:** Procedure of silver recovery from dental amalgam residues using sucrose as reducing agent

	**Dilution**	**Precipitation**		**Reduction**		
**Samplemass (g)**	**32.5% nitric acid volume at room temperature (mL)/ilution**	**20% NaClvolume (mL)**	**Na OH concentration (%)**	**Na OH volume (mL)**	**Sucrose concentration (%)**	**Sucrose volume (mL)**
25.0	30.0/1	60.0	10.0	80.0	2.5	400.0
50.0	50.0/1	100.0	40.0	80.0	20.0	400.0
50.0	60.0/1	120.0	20.0	80.0	5.0	400.0
50.0	100.0/2	100.0	40.0	80.0	20.0	400.0
110.7	300.0/3	500.0	20.0	300.0	20.0	700.0
120.0	300.0/3	500.0	20.0	750.0	20.0	1600.0
134.4	300.0/3	550.0	20.0	400.0	20.0	440.0
150.0	300.0/3	500.0	20.0	750.0	20.0	2400.0
150.0	350.0/4	1200.0	20.0	800.0	20.0	1600.0
150.0	300.0/3	650.0	20.0	500.0	17.5	1100.0
150.0	300.0/3	800.0	20.0	400.0	20.0	2100.0

### Process of silver recovery using ascorbic acid as a reducing agent

**Figure 2:**
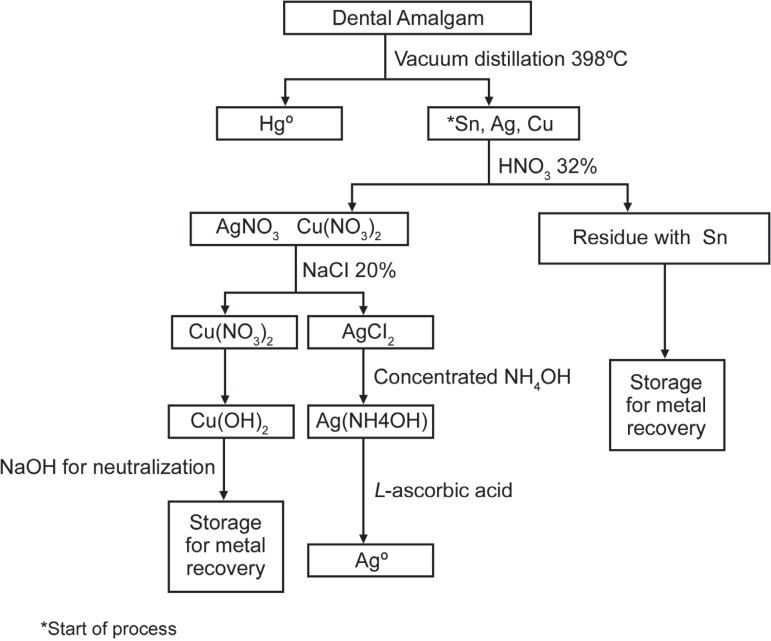
Flow chart of silver recovery using ascorbic acid

The amount of samples used ranged between 150 and 200 g. The tests were done in duplicates, as displayed in Table 2. For dilution, concentrated nitric acid at 90°C was used. The silver nitrate formed was collected in another vial. The precipitation with 20% NaCl was then accomplished as described above. After washing with nitric acid and deionized water (1:100 v/v), NH_3_*H_2_O, water and additional 20% NaCl were added, in order to solubilize the pellet, thus making easier the reaction with ascorbic acid. Ascorbic acid (75-100 g, according to the amount of sample) was then added under agitation and silver was immediately reduced to Ag^0^ (Figure 2).

**Table 2:** Procedure of silver recovery from dental amalgam residues using ascorbic acid as reducing agent

	**Dilution**	**Precipitation**	**Solubilization**			**Reduction**
**Sample mass (g)**	**Concentrated nitric acid volume at 90°C (mL) / dilution**	**20% NaCl volume (mL)**	**Concentrated NH_4_OH volume (mL)**	**Deionized water volume (mL)**	**20% NaCl volume (mL)**	**Amount of ascorbic acid (g)**
A_1_150.0	150.0/1	300.0	500.0	1000.0	-	75.0
A_2_150.0	150.0/1	300.0	500.0	1000.0	-	75.0
B_1_150.0	500.0/5	750.0	200.0	2000.0	1000.0	75.0
B_2_150.0	500.0/5	750.0	200.0	2000.0	1000.0	75.0
C_1_200.0	400.0/3	1200.0	300.0	2000.0	1000.0	100.0
C_2_200.0	400.0/3	1200.0	300.0	2000.0	1000.0	100.0
D_1_200.0	500.0/5	1200.0	300.0	2000.0	1000.0	100.0
D_2_200.0	500.0/5	1200.0	300.0	2000.0	1000.0	100.0
E_1_200.0	400.0/3	1400.0	300.0	2000.0	1500.0	100.0
E_2_200.0	400.0/3	1400.0	300.0	2000.0	1500.0	100.0
F_1_200.0	500.0/5	1400.0	300.0	2000.0	1500.0	100.0
F_2_200.0	500.0/5	1400.0	300.0	2000.0	1500.0	100.0

### Analysis of recovery efficiency

In order to assess the efficiency of recovery, Volhard method was used^19^. Initially, the recovered sample was weighed (±0.01 mg) and then diluted in concentrated nitric acid at 90°C. The resulting solution was titrated with potassium thiocyanate, in the presence of Fe^2+^, that was added as a saturated solution of ammonium ferrous sulfate in 20% nitric acid. In contact with thiocyanate, silver precipitates as AgSCN, which has a very low solubility. A slight excess of thiocyanate is identified by the formation of a soluble ferric complex [FeSCN]^2+^, which is intensively stained in red. The titration error in the Volhard method is small as the indicator is extremely sensitive to the thiocyanate ions. The reaction occurs as described below:

1- AgNO_3_(aq) + KSCN-(aq)→ AgSCN(s) KNO_3_(aq)

2- Fe^3^+(aq) + SCN-(aq) → [FeSCN]^2+^(aq) (dark red ferric complex)

## RESULTS

Table 3 shows the procedures done for silver recovery using sucrose as reducing agent. The recovery (considering that the % of silver in dental amalgam is 32.5%^1^) ranged between 23.5 and 61.6% for the different conditions tested. However, the total time required in the reduction process ranged between 303 and 600 min. The other selected reducing agent was ascorbic acid. The procedures done in the study are described in Table 4. In this case, the reduction occurred immediately, differently from what was seen when the sucrose was used. In addition, the recovery in this case was higher (40% to 95.5%) when compared to the use of sucrose as reducing agent.

**Table 3:** Silver recovery from dental amalgam residues using sucrose as reducing agent

**Sample mass (g)**	**Total time (min)**	**Amount of Agº produced (g)**	**Purity (%)**	**% recovery ª (32.5% of amalgam)**
25.0	303	3.9	99.9	24.4
50.0	430	15.5	49.3	48.4
50.0	360	9.2	94.2	28.8
50.0	373	23.0	87.1	71.9
110.7	390	41.5	52.6	58.6
120.0	330	61.1	44.1	79.6
134.4	455	54.0	19.6	62.8
150.0	600	59.1	84.1	61.6
150.0	440	64.5	86.0	67.2
150.0	365	62.1	52.9	64.7
150.0	565	87.9	37.0	91.6

ª Based on the American Dental Association (ADA) 1 , which considers that the percentage of silver in dental amalgam is 32.5%.

**Table 4:** Procedure of silver recovery from dental amalgam residues using ascorbic acid as reducing

**Sample mass (g)**	**Amount of Agº produced (g)**	**Purity (%)**	**% recovery ª (32.5% of amalgam)**	**Real standard deviation (%)**
A_1_150.0	38.4	99.6	40.0	2.4
A_2_150.0	40.9	97.0	42.6	
B_1_150.0	83.8	78.0	87.3	3.9
B_2_150.0	76.7	80.5	79.9	
C_1_200.0	108.0	91.1	84.4	11.9
C_2_200.0	122.2	67.1	95.5	
D_1_200.0	57.8	99.9	45.2	2.4
D_2_200.0	106.7	52.0	83.4	
E_1_200.0	111.9	72.9	87.4	7.9
E_2_200.0	104.6	69.8	81.7	
F_1_200.0	90.2	82.7	70.5	7.4
F_2_200.0	92.7	89.8	72.4	

ª Based on the American Dental Association (ADA) 1 , which considers that the percentage of silver in dental amalgam is 32.5%.

## DISCUSSION

This is the first study that used ascorbic acid as a reducing agent for recovering silver from dental amalgam. In this study, two methods were tested to recover the silver that is left after the mercury is removed from dental amalgam: one using sucrose as a reducing agent, and the one using ascorbic acid as a reducing agent. The only study in the literature that attempted to recover silver from dental amalgam residues used sucrose as a reducing agent^11^. However, when sucrose was tested as a reducing agent in the present study, the total time required in the reduction process was too long (ranging between 303 and 600 min, depending on the conditions used). Due to this long processing time, a different reducing agent was used in attempt to speed the processing time. The selected reducing agent was ascorbic acid, since it has been reported as a strong reducing agent for metals^17^. In this case, the reduction occurred immediately, differently from what was seen when the sucrose was used. In addition, the recovery was slightly higher when compared to the use of sucrose as reducing agent.

It must be highlighted that some modifications done in the processing technique when the ascorbic acid was used instead of sucrose may have facilitated the reduction process, such as the use of concentrated nitric acid at 90°C, which allowed a faster and more efficient dilution. In the case of sucrose, dilution was performed by the use of 32.5% nitric acid at room temperature. Also the use of NaOH in the case of sucrose leads to the formation of a complex of AgO_2_, which has a very low solubility^20^. Thus, it was necessary to heat this mixture in order to solubilize it, thus demanding a long time for completion of the reducing process (Table 3). In the case of ascorbic acid, NH_4_OH was used instead of NaOH. Thus, a soluble complex is formed and there is more Ag^+^ available to be reduced^20^.

It should also be noted that the procedure of silver recovery using ascorbic acid does not require too much practice, specific knowledge or sophisticated equipments. Thus, its use seems to be viable, due to the high market value of the recovered silver. In addition, the reagents used are not expensive, since for every 150 g of treated silver residues, the cost is approximately around US$ 12.00. The amount of recovered silver is around 65 g, and the cost to buy 1 g of Ag (99% purity) is as high as US$ 2.40. Thus, the recovery of silver using the proposed technique with reduction by ascorbic acid, in addition to reducing the environmental impact caused by the discharge of this metal into the ecosystem, can also provide additional resources to the laboratory that can be used in other investigations.

## CONCLUSIONS

The developed methodology was shown to be viable, since it does not require sophisticated equipments or specialized personnel, and is a cost-effective alternative.
